# DNA Methylation in Ovarian Tumors—a Comparison Between Fresh Tissue and FFPE Samples

**DOI:** 10.1007/s43032-021-00589-0

**Published:** 2021-04-23

**Authors:** Douglas V.N.P. Oliveira, Julie Hentze, Colm J. O’Rourke, Jesper B. Andersen, Claus Høgdall, Estrid V. Høgdall

**Affiliations:** 1 Department of Pathology, Herlev Hospital, University of Copenhagen, Herlev, Denmark; 2 Biotech Research & Innovation Centre, University of Copenhagen, Copenhagen, Denmark; 3 Department of Gynecology, Juliane Marie Centre, Rigshospitalet, University of Copenhagen, Copenhagen, Denmark

**Keywords:** DNA methylation, High throughput, Fresh tissue, FFPE, Ovarian cancer

## Abstract

Among women, ovarian cancer (OC) is one of the most severe forms of malignancy, accounting for a low 5-year survival rate, of approximately 52%. Early symptoms are unspecific and hence hard to detect. The origin of OC and its subtypes are still unclear, underlying the need for efficient diagnostic biomarkers. In that regard, epigenetics studies are emerging in cancer diagnostics, with encouraging outcomes. Among them, DNA methylation profiling has shown that the origins of the cancer epigenome are associated with molecular factors that are crucial to carcinogenesis, such as regulation of oncogenes and tumor suppressors. Furthermore, those events have been detected in abnormal cell morphology before neoplastic formation, indicating its potential crucial use in the OC diagnostics in the future. Nonetheless, studies are limited, and whether methylation analysis can be performed optimally in formalin-fixed paraffin-embedded (FFPE) preparations of OC cases is still elusive. In the present report, we investigated the performance of DNA methylation analysis in FFPE samples, compared to their matched fresh frozen tissue in a small cohort of OC samples. We found that the overall DNA methylation profile in FFPE tissue showed high concordance to that found in fresh frozen tissue, and accounting for the small cohort size, the differentially methylated sites found primarily in frozen tissue, compared to benign samples, were also reproducible in FFPE. Overall, by using samples from our current clinical setting of tissue preservation, these preliminary observations might provide insights into the clinical use of FFPE tissues in methylation studies without critically compromising the outcome.

**Supplementary Information:**

The online version contains supplementary material available at 10.1007/s43032-021-00589-0.

## Introduction

Ovarian cancer (OC) is the second leading cause of death from gynecological malignancy, and the 5-year survival rate is as low as 52% [1]. The major reason for the high mortality is that early symptoms are unspecific, causing more than 60% of patients to be diagnosed in late stages of the disease, where the tumor has spread beyond the pelvic region [1, 2]. The etiology of such disease remains elusive, highlighting the need for efficient biomarkers to support diagnostics and personalized treatment, resulting in better overall patient survival.

Epigenetics is an emerging field in cancer diagnostics. One of the most well-studied epigenetic processes is DNA methylation, characterized by addition of methyl groups to cytosines predominantly in CpG dinucleotide sites. It affects both gene expression and DNA stability [3]. It is an essential process for homeostasis of cellular functions, such as embryogenesis, genome imprinting, and inactivation of chromosome X [4, 5]. In that regard, the origins of the cancer epigenome have been associated with factors that are crucial to embryonic development, constituted massively by hypermethylation of such genes [6, 7]. On the other hand, hypomethylation of oncogene promoters has also been observed in different cancers [8]. Although cancer research in epigenetics has been performed for a few decades, in recent years, the clinical application of cancer-associated methylation profiling has been developing, showing a great potential when it comes to understanding disease development or discovery of new biomarkers, with some promising studies in OC patients [9–13]. Furthermore, as DNA methylation is chemically and biologically stable, methylated sites may have great potential as cancer biomarkers [11, 14–16]. Moreover, because DNA methylation is one the main determinant of cell destination, and consequently cell pathology, this confers a significant advantage of this assay. Among the technologies available, the Infinium® MethylationEPIC BeadChip assay from Illumina is widely used to investigate global methylation patterns. The newest array allows for the investigation of ~850,000 cancer-relevant sites for each individual sample. Within its large coverage, the method includes methylation sites in enhancer regions and gene bodies, bearing the ability to reveal functional sites never investigated before in OC research. For instance, altered methylation patterns have been detected in early stages of different cancer types, even before visible neoplasm [17–19]. Specifically, in OC, the methylation profiling of 27,000 sites suggested that the risk of neoplastic transformation could be predicated in normal cells [18]. Nonetheless, due to the coverage of only 0.1% potential methylation targets in the genome, the investigation of a wider coverage could increase the predictive and diagnostic model needed for clinical application. Therefore, the status of specific methylation sites of the cancer cells can potentially be used to diagnose early stages of cancer.

Formalin-fixed paraffin-embedded (FFPE) tissues are a valuable resource in the clinic, being used in routine diagnostic setting, from immunohistochemistry staining to NGS sequencing. It is the most common form of tissue preservation employed in pathology, whereas storage of fresh frozen tissue has not been implemented to the same extent. Nonetheless, the fact that formalin tends to modify nucleic acids is a concerning caveat for its use in DNA and RNA sequencing-based technologies [20]. However, from extraction and purification protocols to implementation of bioinformatic tools, they are in constant development, seeking to attenuate such issues. From a clinical perspective, it is therefore important to examine DNA methylation in FFPE tissue and whether the results are comparable to those obtained from fresh, untreated tissue. Here, we evaluate the performance of both conservation methods exclusively in OC, employed in our current clinical routine settings. We compared DNA methylation profiles from corresponding FFPE and fresh frozen tissue from the same patients, using the new Infinium® MethylationEPIC BeadChip arrays, by assessing ~850,000 methylation sites. We found that the overall DNA methylation profile in FFPE tissue showed high concordance to that found in fresh frozen tissue. Furthermore, despite the small sample size, the differentially methylated sites found primarily in frozen tissue from OC, in comparison to benign tissue, were also reproducible in FFPE. Overall, by applying our routine clinical setting of tissue storage and preservation, these preliminary findings provide insights into the clinically reproducible use of FFPE tissues in methylation studies, without critically compromising their outcomes.

## Materials and Methods

### Patients

Eight patients were selected from the Pelvic mass/GOVEC cohort, which comprises all women admitted to the Gynecologic Clinic at Rigshospitalet for surgery due to a potentially malignant mass in the pelvic region. Clinical data and treatment information were retrieved from the Danish Gynecologic Cancer Database (DGCD, www.dgcg.dk/) where it is updated continuously.

In total, 3 OC patients with early-stage high-grade serous carcinomas (HGSC) (stages I and II according to FIGO; International Federation of Gynecology and Obstetrics), 3 late-stage OC patients with HGSC (stage III and IV), 1 borderline ovarian tumor of low malignant potential (stage IC1, serous histology), and 1 patient with an ovarian fibroma were included in the study. All patients went through macroradical surgery meaning that none of the patients had received medical treatment before collection of tissue.

### Tissue Samples

FFPE- and fresh frozen tissue, representative of their tumors, were collected from all patients (*n*=8). All tissues were collected during operation, prior to chemotherapy. They were further pathologically verified for histological subtype and grade, and tumor stage was evaluated according to FIGO. All tumor samples were from high-grade serous adenocarcinoma histologic subtype. Samples were registered in the Danish CancerBiobank (Bio- and Genome Bank Denmark) and handled according to their guidelines (www.rbgb.dk). FFPE tissue was kept at room temperature, whereas fresh frozen tissue was stored at −80°C.

### Methylation Analysis

Tissue tumor percentage was scored by a pathologist. FFPE tissue of high tumor percentage was cut out with tissue cylinders of 2 mm, and DNA was extracted using the Maxwell® RSC DNA FFPE Kit (Promega). Fresh frozen tissue was cut with a scalpel in a cold and clean environment and homogenized in TE buffer, before DNA extraction using the Maxwell® RSC Tissue DNA Kit (Promega). All DNA was further purified using the QIAquick PCR purification system (Qiagen) before quantification on a Qubit fluorometer. All DNA samples were diluted to a concentration of 500 ng/μl, and 45 μl was bisulfite-converted using the EZ DNA methylation kit (Zymo Research). DNA purified from FFPE tissue was subsequently repaired using the Infinium HD FFPE DNA Restore Kit (Illumina) and purified using ZR-96 DNA Clean & Concentrator-5 (Zymo Research). Global DNA methylation was profiled with the Infinium® MethylationEPIC BeadChip Kit (manual protocol, Illumina), which covers ~850,000 cancer-related methylation sites. Samples were blinded and randomized before loading on the BeadChip arrays. Stained BeadChips were scanned using the iScan system (Illumina).

### Bioinformatics

Array quality control (QC) assessment, filtering, normalization, and differential methylation analysis were performed in R environment, with IlluminaHumanMethylationEPICanno.ilm10b4.hg19, IlluminaHumanMethylationEPICmanifest, minfi, missMethyl, and DMRcate packages [21–24]. Briefly, QC included removal of probes which presented a detection *P*-value above 0.01 in at least one of the samples. Data were further subjected to functional normalization using quantile normalization, by adjusting for known covariates measuring unwanted variation, specifically designed for Illumina’s platform [25]. Further filtering included removal of probes known to be affected by common SNPs and removal of cross-reactive probes and polymorphic CpGs [26]. *β*-values were calculated as the ratio between the intensities of methylated alleles and total allele intensity. *M*-values were obtained from the logit of *β*-values. Given that *M*-values were reported to be more statistically valid, with a better performance in terms of the ratio between detection rate and true positive rate, we use them in our differential methylation analysis, apart from including the statistics for *β*-values as well for comparison [27].

Comparison of methylation profiles of DNA from FFPE tissue and fresh frozen tissue was done with hierarchical clustering and global correlation analysis, unless otherwise specified.

## Results

**Fig. 1:**
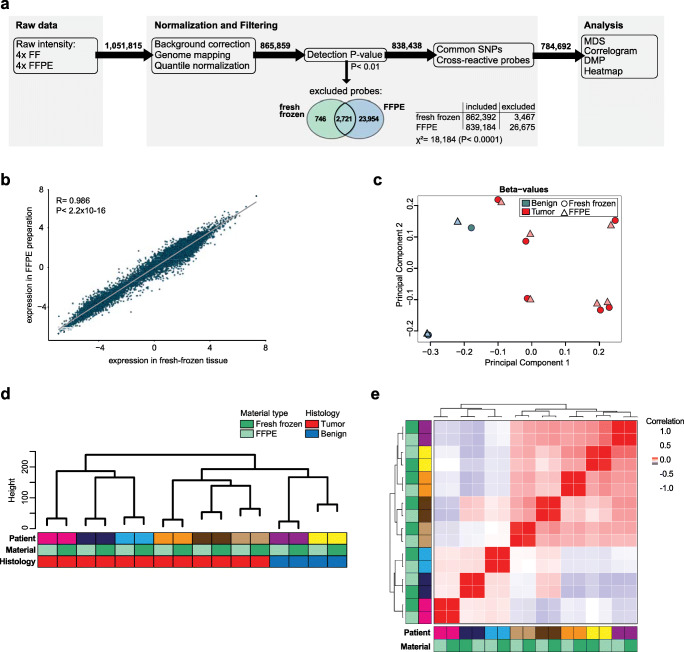
Comparison of DNA methylation profiling in fresh frozen and FFPE-prepared tissue. **a** Workflow analysis for the comparison between tissue preparations. **b** Correlation scatter plot and Pearson’s coefficient of all filtered probes between fresh frozen and FFPE preparations. **c** Principal component analysis (PCA) plot for the most differentially methylated probes of benign (blue) and OC (red) samples, in both fresh frozen (circle) and FFPE (triangle) preparations. **d** Hierarchical clustering for the top 2000 most differentially methylated probes and **e** Correlogram of samples. Dark and light green represent fresh frozen and FFPE preparation, respectively, while different color pairs represent each sample. *P*-values are presented above.

In order to compare the observations in fresh frozen and FFPE tissue, we investigated 865,859 loci across the genome from 2 patients with a benign tumor and 6 malignant paired samples from OC patients. In total, 838,438 (96.8%) probes passed the detection *P*-value threshold (*P*< 0.01), while 27,421 (3.2%) were removed. We further investigated whether the failed probes distributed uniformly on both fresh frozen and FFPE samples. Thus, 87.4% (23,954) were accounted for in the FFPE samples alone, while 2.72% (746) were only in fresh frozen samples, and 9.92% (2,721) in both samples (Fig. 1a). These observations suggest that the quality of FFPE samples is below that of fresh frozen samples (*P*< 0.0001), despite accounting for a minor fraction of the total probes (2.77%).

Further filtering was performed, such as removal of i) probes known to be affected by common SNPs and ii) probes with cross-reactivity in multiple loci in the genome. In total, 784,692 probes were subjected to further analysis (Fig. 1a). First, we evaluated whether those probes would be represented in similar level in both fresh frozen and FFPE samples. Both material types showed a very high concordance in their expression, with *r*^2^=0.986 and *P*-value < 2.2×10^−16^ (Fig. 1b). We next assessed the level of similarity of those samples by performing multidimensional scaling. Data showed that there was no significant difference between fresh frozen and FFPE-paired samples (Fig. 1c). Furthermore, despite the small sample size, this unsupervised classification showed a clear distinction between benign and malignant cases, indicating that there is an overall methylation pattern difference between normal and OC, which was similarly reproduced in both material sources, fresh frozen, and FFPE. Moreover, unsupervised hierarchical clustering was performed in order to evaluate distances between FFPE and fresh frozen samples. To that end, the 2,000 probes with the highest variance across samples were considered. That approach was devised in order to avoid probes with low variance to skew the analysis. Samples from the same patient were highly similar and clustered as expected (Fig. 1d). Moreover, the correlation analysis illustrates the strong association between the DNA methylation profiles of the two tissue types, with correlation coefficients of nearly 1 for FFPE and fresh frozen samples from the same patient (Fig. 1e). The correlation between samples of different tissue types from the same patient was clearly stronger than the correlation between samples of similar tissue-type and histology but from different patients.

**Fig. 2:**
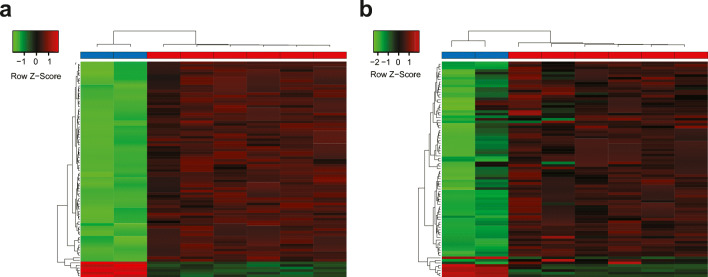
Differentially methylated probes (DMPs) in fresh frozen and FFPE-prepared samples. Differentially expression analysis between benign (blue) and OC (red) samples identified 84 DMPs in fresh frozen tissue (**a**). Those probes were further investigated in FFPE samples (**b**). All probes identified are detailed in the Supplementary table 1

Moreover, we sought to identify differentially methylated probes (DMPs) between benign and malignant fresh frozen samples and further verify whether the candidate targets would be reproducible in FFPE samples. A total of 78 loci were found hypermethylated in tumors, while 6 were hypomethylated in fresh frozen samples (Fig. 2a, Supplementary Table 1). By utilizing those candidates, we further performed the same analysis in FFPE samples. Interestingly, they showed a similar performance in differentiating benign and OC samples (Fig. 2b), further indicating that fresh frozen and FFPE materials present similar methylation patterns. Noteworthy, given the limited number of samples, particularly for the benign cases, these results indicate the reproducibility of those targets in FFPE samples. Thus, a more extensive study with larger cohorts and statistical power is critical in order to confirm these findings. Nonetheless, we have assessed the performance of those 84 candidate DMPs on an external cohort in identifying OC samples, namely, GSE133556 from Gonzalez-Bosquet and colleagues. The cohort is comprised of 12 normal fallopian tubes and 99 high grade serous adenocarcinoma samples. Results showed that those candidates were able to distinguish between the 2 groups (S1 Figure), indicating that the current method has the potential to identify DMPs in FFPE, which could potentially be considered for use in a clinical setting.

## Discussion

Research in cancer etiology and discovery of new biomarkers are important steps towards earlier diagnostics, personalized medicine, and prolonged survival for OC patients. DNA methylation analysis is a robust tool for biomarker discovery and cancer research, but it is key to the analysis that the DNA, including the pattern of methylation, resembles the original tumor from the patient. By tradition, the large majority of tissues collected and stored in hospitals are FFPE. This is still the preferred form of tissue preparation to work with for pathologists in cancer diagnostics, as it allows for a better tissue characterization and tumor classification by traditional procedures, apart from the fact that they can be stored and handled at room temperature. Denmark has established national biobanks, such as the Danish CancerBiobank and Genome Bank, in order to provide the infra-structure for standardized material handling, registration, and storage, including FFPE and fresh frozen tissue from patients. Those national initiatives aim at assuring the high quality of samples to be used in the clinic, as well as research. It has therefore become relevant to test whether methylation analysis should be done preferably in fresh tissue or, if comparable, high quality data can be achieved from FFPE. Our data suggest a very high comparability between global DNA methylation data from fresh frozen tissue and FFPE tissue from ovarian tumors.

Though our results show great similarity for FFPE and fresh frozen tissue, the study can be considered preliminary due to the small patient cohort. However, the consistency of the results, with high correlation between FFPE- and fresh frozen tissue among all patients, suggests that there is a strong correlation between DNA methylation profiles in FFPE and fresh frozen preparations from ovarian tumors, suggesting that the former could be considered in our clinical setting. This was not only true for the 6 HGSC tumors but also for the benign and borderline tumors included in our study, indicating that this could be the general case for ovarian tumors despite disease severity. Similar correlations between fresh frozen tissue and FFPE tissue have been recently reported for brain tumors and breast tumors [28, 29]. Noteworthy, one report has shown that the EPIC platform performs well in fresh frozen and FFPE tissue. However, the assessment was restricted to single samples of each histology and limited to colon, neuron, and renal tissues [30].

The fixation of FFPE tissue is a major advantage in a diagnostic setting as morphological inspection can be performed. The fixed tissue slides can be studied under a microscope, and precise excision of specific tissue is easy. Moreover, the tissue is stable and easy to stack and does not take up freezer space, as it can be scanned or stored in the lab for unlimited time. On the other hand, DNA from fresh tissue is high quality and will always give a more precise picture of the molecular state of the cells as they were in the patient. However, given the current clinical scenario and according to our data, FFPE tissue might be a good alternative to fresh tissue in situations where it is necessary to do DNA methylation analysis and fresh tissue is not available or suitable to work with. Noteworthy, in case of assays for targeted DNA methylation sites used as biomarkers or diagnostic tools, it is always necessary to make a comparison between FFPE- and fresh tissue before choosing FFPE in the routine diagnostic setting.

Though the present data is limited to a few patients, our results indicate possible future use of FFPE for methylation array in the clinic. Before any selected strategy can be decided for routine diagnostic settings, it is necessary to study methylation array analysis on ovarian tumor tissue from larger cohorts. Our study confirms that global DNA methylation data of high quality can be obtained from Illumina Infinium BeadChip array analysis of DNA from ovarian tumors, and it will be highly interesting to see if this technique can be applied to discover new biomarkers for personalized treatment and earlier, life-saving diagnostics for this patient group.

## Supplementary Information

ESM 1 (PDF 449 kb)

ESM 2 (XLSX 14 kb)
