# Supplementary material for: DNA Methylation in Ovarian Tumors—a Comparison Between Fresh Tissue and FFPE Samples
Source: Reprod Sci. 2021 Apr 23;28(11):3212–8. doi: 10.1007/s43032-021-00589-0 (PMC8526488; doi:10.1007/s43032-021-00589-0)
Supplement: Supplementary file 1 — (PDF 449 kb) [file 43032_2021_589_MOESM1_ESM.pdf]

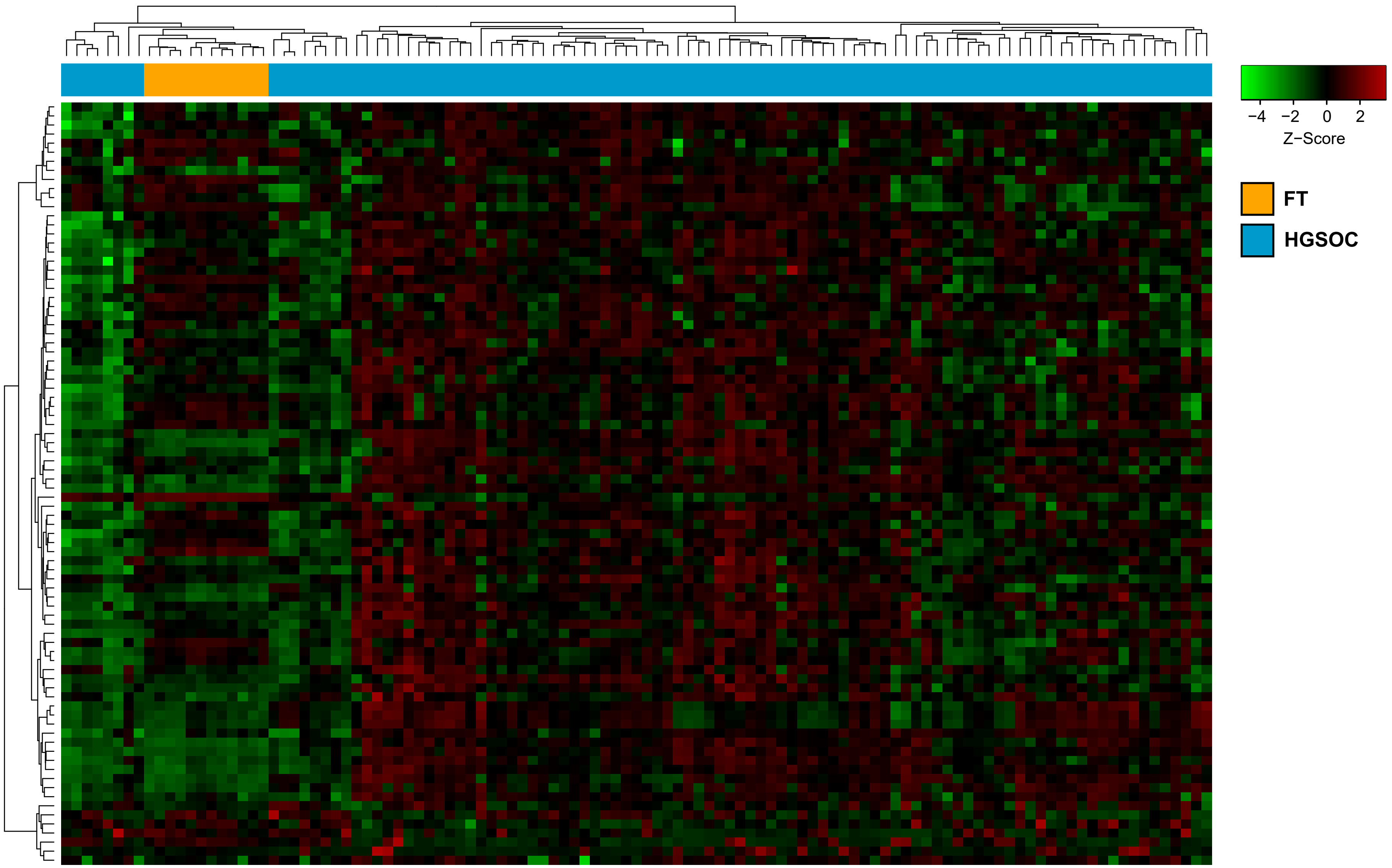

Supplementary Figure S1. Assessment of the 84 DMPs on an independent cohort. Heatmap and hierachical clustering on cohort GSE133556, comprised by 12 samples from Fallopian tube (FT; orange) and 99 samples from high grade serous ovarian carcinoma (HGSOC; blue).
